# Using patient-reported outcome measures to assess psychological well-being in a non-representative US general population during the COVID-19 pandemic

**DOI:** 10.1186/s41687-022-00526-y

**Published:** 2022-11-17

**Authors:** Manraj N. Kaur, Elena Tsangaris, Tanujit Dey, Shelby Deibert, Janina Kueper, Maria Edelen, Andrea L. Pusic

**Affiliations:** 1 Department of Surgery, Patient-Reported Outcomes, Value and Experience (PROVE) Center, Brigham and Women’s Hospital, 75 Francis Street, Boston, MA 02115 USA; 2 Brigham and Women’s Hospital, Harvard Medical School, 75 Francis Street, Boston, MA 02115 USA; 3 Center for Surgery and Public Health, Department of Surgery, Brigham and Women’s Hospital, Harvard Medical School, Boston, MA USA; 4 McMaster University, 1280 Main Street W, Hamilton, ON L8S 4K1 Canada

**Keywords:** Patient-reported outcome measures, Health-related quality of life, Psychological well-being, COVID-19 pandemic

## Abstract

**Purpose:**

The impact of the COVID-19 pandemic on psychological well-being will likely be long-lasting. Efforts directed towards monitoring the onset and progression of distress and mental health disorders are needed to identify and prioritize at-risk populations. This study assesses the psychological well-being of the United States (US) general population during the early phase of the COVID-19 COVID-19 pandemic using validated patient-reported outcome measures (PROMs).

**Methods:**

A cross-sectional study design was used. Adults (18 years or older) who could read and write in English were recruited through Prolific in May 2020. Participants completed a REDCap survey including demographic and health-related questions and three PROMs measuring global health (PROMIS-10 Global Health), anxiety [Generalized Anxiety Disorder Scale-7 (GAD-7)], and depression [Patient Health Questionnaire-9 (PHQ-9)]. A multivariable linear regression was used to identify key factors associated with worse psychological well-being.

**Results:**

Mean age of the 2023 participants was 31.92 ± 11.57 years (range, 18–82). Participants were mainly White (64.7%, n = 1309), female (52.2%, n = 1057), working full-time before the pandemic (43.5%, n = 879), and completed a college, trade, or university degree (40.7%, n = 823). Most participants reported mild to severe anxiety (57.3%, n = 1158) and depression (60%, n = 1276) on the GAD-7 and PHQ-9, respectively. Patient characteristics associated with worse psychological well-being included: age ≤ 39 years, non-White, female or gender diverse, BMI ≥ 30, uninsured, annual income ≤ $49,999 (USD), lower educational attainment, and belief that COVID-19 is deadlier than flu.

**Conclusion:**

PROMs can be used to assess and monitor psychological well-being during the COVID-19 pandemic and to inform the planning and delivery of targeted public health interventions to support at-risk populations.

## Background

The COVID-19 pandemic has emerged as the most pressing public health and economic challenge of our time. As of June 2022, there have been more than 6 million recorded COVID-19-related deaths, with a global economic recession that has surpassed any economic downturn since World War 2. A public health crisis of this magnitude is bound to impact the psychological well-being and health-related quality of life (HRQL) of the masses. Previous research has shown that pandemics result in higher levels of psychological distress, including higher rates of suicide attempts and suicides [1–3]. Many individuals who were previously not considered vulnerable or predisposed to mental illness may experience increased stress levels due to loss of employment, heightened caregiver responsibilities, illness or death of a loved one due to COVID-19, constant media messaging, or distrust of governing bodies [4–6]. In isolation or combined, these factors may result in the onset of new mental health disorders or worsening of pre-existing ones. There is an urgent need to understand the impact of the pandemic and related societal changes on the psychological well-being and overall HRQL of the general population.

Patient-reported outcome measures (PROMs) are questionnaires that assess health status from the patient's perspective [7]. Within healthcare delivery and research, PROMs have been used to understand the outcomes and cost-effectiveness of treatment interventions to improve how healthcare is planned, organized, and delivered. In public health, validated PROMs can be used to assess the psychological well-being and overall health and well-being of the general population during the COVID-19 pandemic. The PROM data when combined with social determinants of health information can be used to target resources and interventions to population subgroups that are most vulnerable to psychological distress. Previous studies have used PROMs to assess the health and well-being of the general population during the early pandemic in countries including China [8–10], the United Kingdom [11, 12], Italy [13], Spain [14], and Brazil [15].

The primary objective of this study was to assess the psychological well-being of the US general population during the COVID-19 pandemic using validated PROMs in the early pandemic (i.e., May 2020). A secondary objective was to examine the relationship between key sociodemographic and clinical variables and psychological well-being.

## Methods

### Ethics

The study was approved by the research ethics board of Mass General Brigham, Boston, Massachusetts (IRB Protocol#: 2020P001440).

### Study design and participants

For this cross-sectional study, participants were recruited through Prolific (Prolific Academic Ltd, Oxford; https://www.prolific.co). Prolific is an online crowdsourcing platform that was established for subject recruitment for research studies. It has a user-friendly interface and includes a minimum payment per unit of time required to complete the study-related task. The participant pool in the Prolific has been shown to be more honest, internationally diverse and less exposed to common research tasks compared to other platforms (e.g., MTurk) [16–18]. A non-representative sample of adult (18 years or older) members of the general public residing in the US at the time of survey administration (i.e., May 2020), who were able to read and write English and did not have cognitive limitations that impacted online survey participation, were included. Eligible participants received an invitation to participate with a brief description of the study objectives and procedures via Prolific's internal email system. Interested participants were asked to click on a link that directed them to a detailed study information sheet. Participants could choose to continue with the survey or ignore the email. Consent was implied if the participant decided to complete the study.

Participants completed an online Research Electronic Data Capture (REDCap) survey hosted at BWH. The survey included questions about participants' sociodemographic characteristics (e.g., age, gender, employment (pre-and post-pandemic)), health status (e.g., smoking status, other pre-existing health conditions), and questions related to their attitudes towards and symptoms of COVID-19. A set of questions (n = 8) derived from a literature review of measures of financial toxicity (e.g., COST [19]) and social determinants of health (SDOH; e.g., Mass General Brigham SDOH questionnaire [20]) that ask about the financial status on a 4-point Likert scale (strongly agree, agree, disagree, strongly disagree) were also included. Participants were asked if they avoided visiting a healthcare service during the pandemic or had a scheduled surgery or a cancelled medical procedure. Female participants were also asked if they were pregnant. Finally, all participants completed three short PROMs, namely the PROMIS-10 Global Health, Generalized Anxiety Disorder Scale-7 (GAD-7), and Patient Health Questionnaire-9 (PHQ-9). The survey took approximately ten minutes to complete, and the participants received monetary compensation (pre-set through Prolific) for their time.

### PROMs administered

#### PROMIS-10 Global Health

The adult PROMIS-10 Global Health (v1.2) short form is a 10-item questionnaire that measures general health and functioning (i.e., overall physical health, mental health, social health, pain, fatigue, and overall perceived quality of life). Evidence suggests that PROMIS-10 Global Health is reliable, valid, and responsive [21–24]. The questionnaire is designed to be applicable across various health conditions. The items in the adult PROMS-10 Global Health are scored on a five-point Likert scale [25, 26] to produce a Global Physical Health and Global Mental Health T-score. High scores reflect more of the concept being measured, i.e., a higher Mental Health score corresponds to better mental health. PROMIS scores have a mean of 50 and a standard deviation of 10 in the US general population. More recently, the cut points or thresholds for the PROMIS-10 Physical Health score categories of excellent, very good, good, fair, and poor have been established [27].

#### Generalized Anxiety Disorder Scale-7 (GAD-7)

The GAD-7 is a 7-item self-report scale developed as a screening tool and severity indicator for generalized anxiety disorder [28]. The items on GAD-7 ask respondents how bothered they are by several anxiety-related problems, over the last two weeks, on a four-point Likert scale (not at all, several days, over half the days, nearly every day). Individual item scores are summed to provide a total score that ranges from 0 to 21, with higher scores indicating more severe generalized anxiety disorder symptoms. Scores of 5, 10, and 15 represent cutpoints for mild, moderate, and severe anxiety, respectively. The psychometric properties of reliability, criterion validity, and construct validity of the GAD-7 have been evaluated in the general population [29], primary care [30], and psychiatric samples [31, 32]. Previous literature suggests that approximately 5% of the general population have GAD-7 scores of 10 or greater, and approximately 1% have GAD-7 scores of 15 or greater [28].

#### Patient Health Questionnaire-9 (PHQ-9)

The PHQ-9 is a nine-item questionnaire designed as a screening tool for depression in primary care and other healthcare settings. Each question asks the frequency that a patient has experienced a particular depressive symptom in the past two weeks on a four-point Likert scale (not at all, several days, more than half the days, nearly every day). The responses on the items are totalled and range from 0 to 27, with higher scores indicating more severe depression. Scores of 5, 10, 15, and 20 represent cutpoints for mild, moderate, moderately severe and severe depression, respectively. The PHQ-9 has been widely validated and has shown good psychometric properties against other established and commonly used PROMs for depression assessment, such as the Beck Depression Inventory (BDI) and the Hamilton Depression Rating Scale (HDRS) [33]. The PHQ-9 is shorter (9 questions) as compared to BDI and HDRS which have 21 and 17 questions respectively and is recommended by the American Academy of Family Physicians as a tool for depression screening. Previous research has established that women tend to score 3.1 points higher than men and that 18.1% of the population will have mild depression (PHQ score, 5–9), and 8.1% will have moderate to severe depression (PHQ score, 10–27) [30, 34].

### Statistical analysis

All statistical analysis was performed using IBM SPSS Statistics for Mac Version 26.0 (IBM Corp. Released 2019. Armonk, NY: IBM Corp) and R software, version 4.0.2 [35]. A sample size of 2000 participants was set a priori and was based on the budgetary constraints of the study. No sample size calculation was performed. However, due to a glitch in the system, 2023 participants responded to the survey. Data from all 2023 participants were included in the analysis. Means and standard deviations were used to summarize the PROM data by the demographic and clinical characteristics of the participants. Categorical variables were summarized using frequencies and percentages, and contingency tables were used to assess the distribution of the PROM scores by demographic and clinical variables. A one-way analysis of variance was conducted to compare the 8 financial questions with the 4 PROM scores.

A set of key demographic and clinical factors were selected based on clinical reasoning and the literature [36–49] and their relationship was examined with the 4 PROM scores (i.e., PROMIS-10 Global Mental Health T-score, PROMIS-10 Global Physical Health T-score, GAD-7 score, and PHQ-9 score) with a multivariable linear regression analysis. The variables included in the regression were age, gender, ethnicity, BMI, education attainment, marital status, living arrangement, income and insurance status, prior psychiatric illness, and the belief that COVID-19 is deadlier than flu. For the regression analysis, the BMI variable was recoded as normal or overweight (BMI < 25–29.99) and obese (BMI ≥ 30), ethnicity was recoded as White and Others, marital was recoded as married or living common-law and Others, the living arrangement was recoded as live alone or live with someone, and the highest level of education was recoded as low (i.e., some or completed high school or some college, trade or university diploma) and high (completed college, trade or university diploma and post-graduate degree). The income variable was recoded to ≤ $49,999 and ≥ $50,000 and the insurance variable was recoded to uninsured and insured. The statistical significance was set at *p* value < 0.05.

## Results

The mean age of the 2023 participants was 31.92 years (SD, 11.57; range 18 to 82 years), and the mean body mass index (BMI) was 25.58 kg/m^2^ (SD, 6.13, range 10.38 to 55.22). Majority of the participants were White (n = 1,309, 65%), young adults (n = 1,589, 79%), single or never been married (n = 1,188, 59%), had completed college, trade or university (n = 823, 41%), and reported no change in employment due to COVID-19 (n = 1,382 m 68%). Further, most of the participants reported no pre-existing mental (n = 1,428, 71%), cardiorespiratory (n = 1,847, 91%), cancer (n = 1,992, 99%), diabetes (n = 1,968, 97%), blood-related (n = 1,841, 91%), autoimmune (1,911, 95%) illness. A total of 13 (< 1%) participants reported that they had tested positive for the COVID-19 and 17 (< 1%) participants reported living with someone who had tested positive for the COVID-19. Most participants believed COVID-19 virus was more deadly than flu (n = 1,574, 78%) and said that they were planning to receive the vaccine when one becomes available (n = 1,340, 66%).

The mean scores for PROMIS-10 Global Physical and Mental Health, GAD-7 and PHQ-9 by the demographic and clinical characteristics of the participants are provided in Table 1. The mean PROMIS-10 Global Physical Health T-scores for most of the patient characteristics were found to be close to the reference value for the US general population of 50; however, the mean Mental Health T-scores were much lower (i.e., worse mental health compared to the general population) across several patient characteristics. This deviation from the reference value of 50 for the Mental Health T-score was much more pronounced for participants who chose “other” as gender, were Indigenous, lived alone, had some high school education, had a change in the type of employment, reported less than USD 15,000 annual income in the previous year, were uninsured, had a pre-existing chronic or mental health condition, and had their scheduled surgery cancelled due to the pandemic. For the GAD-7 and PHQ-9, participants who identified as the “other” gender or Indigenous, had lost their job or were unable to work, were uninsured, and had their surgery cancelled reported higher levels of anxiety and depression, respectively.

**Table 1:** Mean and standard deviation scores for PROMIS-10 physical health, mental health, GAD-7 and PHQ-9 by participant demographic and clinical characteristics

Category	Sub-category	n	%	PROMIS-10 physical health T-scores		PROMIS-10 mental health T-scores		GAD-7		PHQ-9	
				Mean	Standard deviation	Mean	Standard deviation	Mean	Standard deviation	Mean	Standard deviation
US region	Northeast	384.00	19.00	50.19	7.54	44.76	8.93	6.85	5.37	7.61	6.09
	Midwest	352.00	17.40	50.50	8.27	45.83	8.98	6.34	5.18	7.04	5.77
	South	724.00	35.80	50.10	7.82	45.63	9.01	6.42	5.41	7.24	5.94
	West	563.00	27.80	50.24	7.64	45.60	8.24	6.22	5.17	7.00	5.69
Gender	Male	945.00	46.70	51.24	7.54	46.84	8.84	5.33	4.78	6.24	5.50
	Female	1057.00	52.30	49.40	7.93	44.52	8.45	7.33	5.52	7.97	6.02
	Others	21.00	1.00	46.20	6.21	33.85	8.12	10.81	5.78	12.57	6.06
Ethnicity	White/Caucasian	1309.00	64.70	50.35	7.91	45.74	8.97	6.54	5.35	7.14	5.89
	Black/African American	157.00	7.80	50.22	8.09	44.20	9.14	5.90	5.38	6.55	5.81
	Hispanic/Latino	163.00	8.10	50.56	7.71	46.45	8.22	6.34	5.74	7.79	6.30
	Asian/Pacific Islander	313.00	15.50	50.02	6.85	45.35	7.66	5.91	4.60	7.15	5.23
	Indigenous / Aboriginal	9.00	0.40	43.89	9.43	42.12	5.39	10.89	6.17	12.44	8.03
	Others	72.00	3.50	48.87	8.64	42.61	9.96	7.44	5.54	8.22	6.48
Age	Young adults (≤ 38.9 years)	1589.00	78.50	50.27	7.57	45.12	8.72	6.70	5.34	7.49	5.92
	Middle-aged adults (39–59.9 years)	366.00	18.10	50.02	8.41	46.47	8.66	5.61	5.02	6.36	5.57
	Old adults (≥ 60 years)	68.00	3.40	50.23	9.39	48.86	9.83	4.65	5.12	5.29	5.48
BMI	Underweight/normal (< 24.9)	1128.00	55.80	51.36	7.65	45.92	8.81	6.43	5.25	7.06	5.70
	Overweight (25–29.9)	492.00	24.30	50.55	7.52	46.38	8.58	5.77	4.94	6.53	5.68
	Obese (≧ 30)	389.00	19.20	46.71	7.55	43.13	8.59	7.33	5.78	8.52	6.42
Marital Status	Single, never married	1188.00	58.70	50.00	7.54	44.18	8.55	6.67	5.19	7.83	5.88
	Living common law	114.00	5.60	48.27	7.57	45.42	7.32	7.15	5.37	7.55	5.59
	Married	554.00	27.40	51.77	7.77	48.91	8.35	5.41	5.22	5.47	5.32
	Widowed/divorced/separated	123.00	6.10	48.47	9.21	43.97	9.94	7.18	5.98	8.09	6.83
	Others	44.00	2.20	46.95	7.58	42.35	8.67	8.84	5.13	9.05	5.56
Living arrangement	Alone	355.00	17.50	49.87	7.87	44.45	8.74	6.43	5.37	7.67	6.15
	With 1 other person	479.00	23.70	50.24	8.00	45.63	8.52	6.72	5.38	7.13	5.80
	With 2 other people	418.00	20.70	49.90	7.74	45.14	8.71	6.59	5.37	7.36	5.73
	With 3 other people	405.00	20.00	51.30	7.69	46.62	9.24	5.82	5.16	6.45	5.63
	With 4 other people	238.00	11.80	49.61	7.07	45.22	8.16	6.78	5.10	7.80	6.04
	With 5 or more people	128.00	6.30	49.98	8.37	45.98	9.46	6.12	5.28	7.05	6.00
Live with children under 16 years	Yes	345.00	86.50	51.27	8.46	48.20	8.82	5.56	5.36	5.71	5.59
	No	54.00	13.50	50.36	8.82	48.70	8.39	4.93	4.99	5.54	6.05
Highest level of education	Some high school	29.00	1.40	49.19	8.60	43.82	10.09	6.24	5.72	8.41	7.20
	Completed high school	230.00	11.40	49.32	7.73	42.92	9.54	7.23	5.50	8.62	6.27
	Some college, trade, or university diploma	518.00	25.60	49.53	7.85	44.43	8.50	6.84	5.40	7.91	6.28
	Completed college, trade, or university diploma	823.00	40.70	50.43	8.00	46.20	8.79	6.16	5.31	6.66	5.60
	Some Masters/doctoral degree	99.00	4.90	50.70	6.71	46.54	6.92	6.19	5.35	7.51	5.75
	Completed Masters/doctoral degree	324.00	16.00	51.42	7.30	47.04	8.48	5.98	4.83	6.28	5.11
Employment change due to COVID-19	Yes	641.00	31.70	49.62	7.58	44.43	8.11	7.49	5.47	8.32	5.97
	No	1382.00	68.30	50.51	7.88	45.98	9.04	5.94	5.15	6.69	5.75
Type of change in employment due to COVID-19	Reduced paid hours (e.g., Full time to part-time)	69.00	3.40	50.12	8.39	44.82	7.80	7.52	5.47	8.04	6.18
	Increase in paid hours (e.g., Part-time to Full-time)	10.00	0.50	48.27	4.49	45.88	7.38	5.70	3.83	5.90	5.30
	Change in type of employment (e.g., employed to self-employed)	18.00	0.90	46.79	8.01	42.99	10.35	7.44	7.04	8.56	6.53
	Lost job/unable to work/retired	293.00	14.50	49.06	7.48	43.64	8.31	8.16	5.50	9.14	6.08
Front-line worker	Yes	41.00	2.00	50.14	7.58	45.91	8.43	6.22	5.27	6.68	5.91
	No	1982.00	98.00	50.46	7.43	45.38	7.95	6.78	5.30	7.40	5.69
Total household income in previous year	Less than $15,000	213.00	10.50	47.38	7.82	42.22	8.65	7.61	5.73	8.77	6.39
	$15,000 to $24,999	199.00	9.80	48.08	8.40	43.04	9.06	7.35	5.60	8.67	6.40
	$25,000 to $49,999	415.00	20.50	49.41	7.46	44.36	8.55	6.65	5.54	7.57	6.00
	$50,000 to $75,000	432.00	21.40	50.84	7.91	46.42	8.25	6.13	5.16	6.55	5.58
	> $75,000	664.00	32.80	51.68	7.34	47.13	8.70	5.92	4.95	6.57	5.50
Current health insurance status	Uninsured	274.00	13.50	49.33	7.82	43.47	8.66	7.06	5.56	8.16	5.91
	Insured	1520.00	75.20	50.59	7.73	46.19	8.69	6.15	5.15	6.79	5.71
	Other	229.00	11.30	48.38	8.06	43.07	8.95	8.02	5.91	9.53	6.85
Smoking status	Currently smoking	176.00	8.70	48.01	9.02	44.81	9.87	6.99	5.67	8.49	6.74
	Recently quit	204.00	10.10	47.75	8.11	42.80	9.08	7.32	5.67	8.47	6.44
	Never smoked	1643.00	81.20	50.77	7.51	45.90	8.56	6.26	5.20	6.92	5.66
Mental health condition	Yes	595.00	29.40	46.94	7.74	40.41	8.02	9.46	5.46	10.55	6.25
	No	1428.00	70.60	51.60	7.40	47.61	8.20	5.17	4.69	5.82	5.10
Cardiorespiratory condition	Yes	176.00	8.70	46.21	8.51	43.45	9.53	7.87	5.71	8.45	6.15
	No	1847.00	91.30	50.61	7.61	45.69	8.69	6.29	5.24	7.09	5.83
Cancer	Yes	31.00	1.50	47.07	10.77	48.17	8.65	4.71	4.84	6.23	6.59
	No	1992.00	98.50	50.28	7.73	45.45	8.78	6.46	5.30	7.22	5.86
Diabetes	Yes	55.00	2.70	45.10	8.62	47.68	8.36	5.95	5.54	7.13	6.20
	No	1968.00	97.30	50.37	7.72	45.43	8.79	6.44	5.29	7.21	5.86
Blood-related condition	Yes	182.00	9.00	45.96	8.62	42.84	9.12	7.91	5.53	8.96	6.39
	No	1841.00	91.00	50.65	7.58	45.75	8.71	6.28	5.25	7.04	5.79
Autoimmune disease	Yes	112.00	5.50	44.56	9.19	42.15	8.99	8.55	5.91	9.86	6.34
	No	1911.00	94.50	50.56	7.58	45.69	8.73	6.31	5.24	7.05	5.80
Received organ transplant	Yes	2.00	0.10	47.55	14.35	49.55	1.77	3.50	4.95	6.00	5.66
	No	2021.00	99.90	50.23	7.79	45.49	8.79	6.43	5.30	7.21	5.87
Cancelled elective surgery	Yes	40.00	2.00	44.35	9.97	40.53	9.73	10.53	6.33	10.97	8.05
	No	1983.00	98.00	50.34	7.70	45.59	8.74	6.35	5.25	7.14	5.80
Pregnant	Yes	16.00	1.50	50.29	6.28	47.42	7.66	5.00	4.93	5.00	3.95
	No	1041.00	98.50	49.39	7.95	44.47	8.45	7.36	5.52	8.01	6.04

The mean scores and distribution by the 3 PROMs based on the responses to the question on self-reported financial status are shown in Table 2. Most participants in the sample reported being able to meet their monthly expenses (86%, n = 1,736); however, a substantial number of participants had to cut down on expenses (64%, n = 1,296), were not happy with their current financial situation (54%, n = 1,089), and reported worrying about their future financial status (72%, n = 1,457). Similar to the demographic and clinical variables, the mean value for the PROMIS-10 Global Physical Health was close to the reference value of 50, whereas larger variability was noted for the PROMIS-10 Global Mental health scores for the financial questions. A one-way analysis of variance analysis revealed that there was a statistically significant difference in the mean PROM scores and all 8 questions (*p* < 0.001). Post-hoc analysis using Tukey’s HSD test for multiple comparisons found that the mean value of the 4 PROM scores was significantly different except between response levels disagree and strongly disagree for questions asking about the ability to meet monthly expenses, money saved for essentials, feeling financially stressed, and feeling worried about future financial status; between strongly agree and agree for questions asking about the current financial situation, having to cut down on expenses and the need to borrow money; and between strongly agree, agree and disagree for the question asking about borrowing money from a financial institution.

**Table 2:** Mean and standard deviation scores for PROMIS-10 physical health, mental health, GAD-7 and PHQ-9 by answers to questions about financial status

Question about financial status	Response categories	N	%	PROMIS-10 physical health T-scores		PROMIS-10 mental health T-scores		GAD-7		PHQ-9	
				Mean	Standard deviation	Mean	Standard deviation	Mean	Standard deviation	Mean	Standard deviation
I am able to meet my monthly expenses	Strongly agree	628	31.04	52.79	7.36	49.07	8.59	4.91	4.70	5.41	4.92
	Agree	1108	54.77	49.84	7.36	44.91	8.01	6.45	5.18	7.22	5.75
	Disagree	228	11.27	46.21	8.29	40.19	8.20	9.09	5.24	10.78	6.10
	Strongly disagree	59	2.92	45.66	8.37	38.91	10.16	11.95	6.08	12.29	7.23
I am happy with my current financial situation	Strongly agree	214	10.58	53.99	8.02	52.12	8.74	4.19	4.83	4.68	4.71
	Agree	720	35.59	51.83	6.98	48.06	7.65	4.97	4.58	5.58	5.21
	Disagree	757	37.42	49.15	7.48	43.77	7.61	7.00	5.01	7.81	5.46
	Strongly disagree	332	16.41	46.77	8.15	39.57	8.84	9.77	5.82	11.01	6.63
I have enough money saved to cover cost of essentials (food, rent, medications)	Strongly agree	544	26.89	52.91	7.57	49.42	8.55	4.88	4.56	5.38	4.72
	Agree	1015	50.17	50.38	7.11	45.28	7.96	6.24	5.09	6.94	5.59
	Disagree	300	14.83	46.92	7.84	41.73	8.54	8.39	5.77	9.55	6.38
	Strongly disagree	164	8.11	46.41	8.71	40.64	9.15	9.23	5.82	10.65	7.12
I have had to cut down on expenses	Strongly agree	440	21.75	48.28	8.06	43.62	8.77	8.09	5.74	8.78	6.11
	Agree	856	42.31	49.54	7.61	44.54	8.39	6.81	5.10	7.73	5.99
	Disagree	583	28.82	51.80	7.17	46.96	8.56	5.26	4.99	5.91	5.25
	Strongly disagree	144	7.12	53.87	8.10	50.91	8.97	3.89	4.18	4.60	4.78
I need to borrow money from a friend, family member, relative, colleague	Strongly agree	120	5.93	46.55	8.00	40.75	9.48	10.27	5.91	11.53	6.45
	Agree	277	13.69	47.21	7.90	42.32	8.23	8.36	5.08	9.79	5.92
	Disagree	806	39.84	49.96	7.45	44.97	8.09	6.16	5.13	6.83	5.71
	Strongly disagree	820	40.53	52.04	7.52	47.77	8.84	5.48	5.02	6.08	5.36
I need to borrow money from bank or other financial institution	Strongly agree	66	3.26	47.01	9.24	43.18	10.42	9.65	6.57	10.00	6.79
	Agree	182	9.00	47.50	7.02	42.42	7.79	8.66	5.20	10.08	5.94
	Disagree	840	41.52	49.68	7.63	44.50	8.39	6.45	5.21	7.14	5.88
	Strongly disagree	935	46.22	51.48	7.72	47.15	8.86	5.75	5.10	6.52	5.55
I feel financially stressed	Strongly agree	384	18.98	46.30	8.24	40.44	8.71	10.07	5.81	11.02	6.45
	Agree	684	33.81	49.22	7.10	43.70	7.67	7.20	4.73	7.95	5.49
	Disagree	696	34.40	51.85	6.96	47.72	7.82	4.77	4.62	5.44	5.04
	Strongly disagree	259	12.80	54.34	7.84	51.73	8.50	3.47	3.97	4.40	4.55
I worry about my future financial status	Strongly agree	538	26.59	46.83	7.95	40.61	8.36	9.70	5.59	10.66	6.17
	Agree	919	45.43	50.01	7.00	45.21	7.65	6.32	4.66	7.06	5.33
	Disagree	417	20.61	53.40	6.88	49.75	7.51	3.59	3.96	4.28	4.46
	Strongly disagree	149	7.37	54.93	8.41	52.93	9.19	3.30	4.50	3.86	4.63

Table 3 show the results of the regression analysis. We found that younger adults scored worse on the PROMIS-10 Mental health score, GAD-7 and PHQ-9, and the difference between younger and older adults was significant. The younger adults had higher scores on the Global 10 Physical health score, indicating better physical well-being. Individuals who identified as females and chose others as their gender response had worse psychological being compared to men. Women on average scored 3 and 3.5 points higher on the GAD-7 and PHQ-9 respectively. Similarly, individuals who had BMI ≥ 30 had worse outcomes on all 4 PROM scores. Participants from non-White ethnic backgrounds scored worse compared to White participants on PROMIS-10 Physical and Mental scores but not with GAD-7 or PHQ-9. Lower income (i.e., ≤ $49,999), being uninsured, and having a history of prior psychiatric illness were associated with statistically significant worse psychological well-being as compared to income ≥ $50,000, being insured and having no history of prior psychiatric illness. Participants who were separated, divorced, or widowed scored had worse Global 10 Mental Health and PHQ-9 scores compared to individuals who were married or common-law, whereas living arrangement was not associated with any of the 4 PROM scores. The belief that COVID-19 was deadlier than flu was associated with worse outcomes on all 4 PROMs.

**Table 3:** Regression analyses results

Variable	PROMIS Global 10-physical score		PROMIS Global 10-mental score		GAD-7		PHQ-9	
	Co-efficient (95% CI)	*p* value	Co-efficient (95% CI)	*p* value	Co-efficient (95% CI)	*p* value	Co-efficient (95% CI)	*p* value
Age (reference: young adults)								
Middle-aged adults	− 0.13 (− 1.0, 0.75)	0.77	0.52 (− 0.42, 1.5)	0.28	− 1.2 (− 1.7, − 0.57)	**<** **0.001**	− 0.87 (− 1.5, − 0.22)	**0.008**
Older adults	− 0.37 (− 2.2, 1.4)	0.68	2.4 (0.48, 4.3)	**0.014**	− 1.7 (− 2.9, − 0.54)	**0.004**	− 1.6 (− 2.9, − 0.24)	**0.020**
Gender (reference: male)								
Female	− 1.0 (− 1.7, − 0.38)	**0.002**	− 1.6 (− 2.3, − 0.86)	**<** **0.001**	1.5 (1.1, 1.9)	**<** **0.001**	1.2 (0.74, 1.7)	**<** **0.001**
Other	− 2.6 (− 5.7, 0.60)	0.11	− 8.4 (− 12, − 5.0)	**<** **0.001**	2.9 (0.79, 5.0)	**0.007**	3.5 (1.2, 5.8)	**0.003**
BMI (reference: BMI ≤ 29.99)								
Obese (BMI ≥ 30)	− 3.7 (− 4.5, − 2.9)	**<** **0.001**	− 2.1 (− 3.0, − 1.2)	**<** **0.001**	0.61 (0.07, 1.2)	**0.028**	1.1 (0.49, 1.7)	**<** **0.001**
Ethnicity (reference: White)								
Non-White	− 1.0 (− 1.7, − 0.31)	**0.005**	− 0.91 (− 1.6, − 0.17)	**0.016**	− 0.11 (− 0.58, 0.35)	0.63	0.37 (− 0.14, 0.88)	0.15
Marital status (reference: married/living common-law)								
Others (e.g., single, divorced, widowed, separated)	− 0.48 (− 1.3, 0.32)	0.24	− 2.6 (− 3.5, − 1.8)	**<** **0.001**	0.40 (− 0.13, 0.94)	0.14	1.0 (0.43, 1.6)	**<** **0.001**
Living arrangement (reference: live alone)								
Live with someone	− 0.39 (− 1.3, 0.54)	0.41	0.08 (− 0.91, 1.1)	0.88	0.00 (− 0.61, 0.62)	0.99	− 0.26 (− 0.94, 0.42)	0.46
Education attainment (reference: Low—less than college, trade or university diploma)								
High—completed college, trade, university diploma or higher	0.51 (− 0.18, 1.2)	0.14	1.1 (0.38, 1.9)	**0.003**	− 0.44 (− 0.89, 0.02)	0.063	− 0.85 (− 1.4, − 0.34)	**<** **0.001**
Annual Income (reference: ≤ $49,999)								
≥ $50,000	1.8 (1.2, 2.5)	**<** **0.001**	1.6 (0.86, 2.3)	**<** **0.001**	− 0.47 (− 0.93, − 0.01)	**0.044**	− 0.56 (− 1.1, − 0.05)	**0.030**
Insurance status (reference: uninsured)								
Insured	1.1 (0.37, 1.9)	**0.004**	1.8 (1.0, 2.6)	**<** **0.001**	− 0.92 (− 1.4, − 0.41)	**<** **0.001**	− 1.3 (− 1.8, − 0.70)	**<** **0.001**
Prior psychiatric illness (reference: no prior psychiatric illness)								
Prior psychiatric illness	3.9 (3.1, 4.6)	**<** **0.001**	6.2 (5.4, 6.9)	**<** **0.001**	− 3.7 (− 4.1, − 3.2)	**<** **0.001**	− 4.1 (− 4.6, − 3.6)	**<** **0.001**
Believe that COVID-19 is deadlier than flue (reference: believe COVID-19 is not deadlier than flu)								
Believe COVID-19 is deadlier than flu	1.2 (0.39, 1.9)	**0.003**	1.6 (0.75, 2.4)	**<** **0.001**	− 1.0 (− 1.5, − 0.45)	**<** **0.001**	− 1.1 (− 1.6, − 0.50)	**<** **0.001**

Bold indicates significant *p* value; CI, confidence interval

## Discussion

In this cross-sectional survey of the US general population, we found that valid, reliable, and generic PROMs can be used to assess psychological well-being and health-related quality of life during the COVID-19 pandemic. Individuals who were female or other gender, younger, non-White, obese, not married or living common-law, uninsured, diagnosed with psychiatric illness, and earned less than $49,999 annual income in the prior year were at a higher risk of poor psychological well-being.

Recent literature on the psychological well-being of populations during the pandemic corroborates our findings [38–40, 42, 50, 51]. Females have been found to have worse psychological well-being compared to males, possibly due to a couple of reasons. First, females are more likely to work in industries, such as retail, that were more negatively affected by the pandemic. Further, women may be working "second shifts" as primary caregivers to children, elderly or infected family members. We also found that young to middle-aged adults experienced higher psychological distress compared to their older counterparts, which may be associated in part with job loss and financial uncertainty. Further, this group is more likely to consist of digitally literate individuals and hence, are subject to distress related to media messaging in the context of COVID-19 [5, 6, 52, 53]. Lastly, our findings align with previous research that suggests poor economic status, lower education level, and being uninsured may cause individuals to develop new mental illness(es), especially depression, during the pandemic [5, 54–56].

We found that there was a statistically significant difference in the mean scores of the 4 PROMs and the 8 questions asking about financial status. A post hoc analysis for multiple comparisons revealed that the levels at which this difference occurred were not consistent. This is an interesting but also a non-informative finding of this study. The financial status questions were included to get a sense of the participant’s financial status and the source of distress related to aspects of financial status (e.g., being able to meet monthly expenses, borrowing from financial institutions); and were derived from pre-existing measures of financial toxicity and social determinants of health. The response levels were determined by the study team. As the intention was not to develop a new scale to assess financial status during the pandemic, an a priori evaluation of the questions and the construct measured was not completed (e.g., exploratory factor analysis). A plausible cause for the inconsistency noted in post hoc group comparisons is that the response levels for the financial questions were unable to adequately discriminate between the participants. Subsequently, we chose to use the most used indicator of annual income in our regression model and not the responses to these individual questions or a make-do summed score. However, these results underline the need for assessing the financial status during the pandemic (especially, in relation to psychological well-being) using validated measures.

**Fig. 1:**
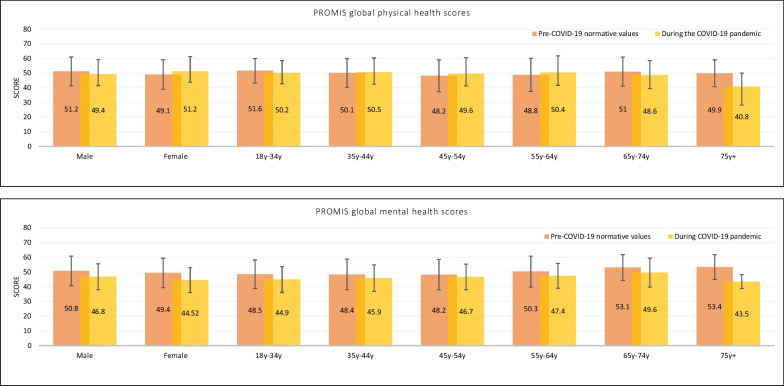
PROMIS global physical and mental health T-scores—reference scores versus scores during the pandemic

A post hoc descriptive comparison of published reference values for the PROMs from pre-pandemic literature with our study showed a higher prevalence of anxiety and depression in the general population during the pandemic than the pre-pandemic levels. The T-scores for the PROMIS-10 Global Physical and Mental Health scales were found to be lower than the reference values during the pandemic for all population subgroups by gender and age; however, for physical health, the T-scores were slightly better than the reference values for females and individuals between the ages of 45–64 years (Table 4, Fig. 1). For the GAD-7, 25.2% (n = 510) of the participants reported scores of 10 or higher, and 9.3% (n = 189) reported scores of 15 or higher. On PHQ-9, women scored 1.73 points higher than men, with 29.2% (n = 590) reporting mild depression and 31.1% (n = 630) reporting moderate to severe depression. However, these results are merely conjecture due to small sample sizes in some of the categories of reference groups and the unrepresentativeness of our sample. Future studies should explore the shift in reference values during the pandemic and how long the response shifts last post-pandemic.

**Table 4:** Normative values for the US general population for the PROMIS Global Health measure

Category	Normative values		COVID-19 study	
	Score	N	Score	N
(a) *PROMIS global mental health*				
Male	50.8 ± 10.0	2206	46.8 ± 8.8	945
Female*	49.4 ± 10.0	3008	44.52 ± 8.4	1057
18–34 years	48.5 ± 9.7	1183	44.9 ± 8.7	1357
35–44 years	48.4 ± 10.4	863	45.9 ± 9.0	376
45–54 years	48.2 ± 10.3	902	46.7 ± 8.7	169
55–64 years	50.3 ± 10.5	873	47.4 ± 8.4	90
65–74 years	53.1 ± 8.8	715	49.6 ± 9.8	28
75 years+	53.4 ± 8.4	679	43.5 ± 4.7	3
(b) *PROMIS global physical health*				
Male	51.2 ± 9.8	2212	49.4 ± 7.9	945
Female*	49.1 ± 10.1	3015	51.2 ± 7.5	1057
18–34 years	51.6 ± 8.4	1182	50.2 ± 7.5	1357
35–44 years	50.1 ± 9.8	865	50.5 ± 8.0	376
45–54 years	48.2 ± 10.9	910	49.6 ± 8.3	169
55–64 years	48.8 ± 11.3	875	50.4 ± 8.8	90
65–74 years	51.0 ± 9.9	713	48.6 ± 9.2	28
75 years +	49.9 ± 9.2	683	40.8 ± 12.6	3

*The gender category does not add up to 2023 because participants who chose “other” are not shown in this table

The study results have important implications. First, this study demonstrates that PROMs can be used to screen and monitor psychological well-being during a pandemic. When linked with other relevant public health data, such as sociodemographic information, healthcare usage, and morbidity outcomes by zip code, PROMs can play a critical role in achieving effective and efficient healthcare delivery by targeting the health resource allocation to the most vulnerable subgroups of the population. Additionally, PROMs can be used to assess the quality of life impact of "long COVID-19 [57, 58]” during hospitalization to enhance the management of patients, support discharge pathways during recovery and survivorship, and determine unmet needs [59, 60]. Finally, this study highlights the importance of asking about stressors (e.g., change in employment status, front-line worker) in the context of COVID-19 when conducting assessments of psychological well-being.

Our study has some important limitations. An online crowdsourcing platform was used for recruitment purposes, which may have resulted in the exclusion of population subgroups that are digitally illiterate, have no or inadequate access to technology and the internet, and non-English speaking. Further, we did not pre-set recruitment quotas for gender, ethnicity or age groups, resulting in a non-representative sample of the US population. To put this in perspective, according to the recent US Census data (https://www.census.gov/quickfacts/fact/table/US/PST045221), 16.5% of the adults are 65 years and older, 32.9% have college or higher level of education, 50.8% identify as females and 76.3% are White. The study sample included 1.5% of adults that were 65 years and older, 61.6% had college or higher level of education, 52.3% were females and 64.7% were White. The under-representation of these subgroups is an important limitation since the negative psychological well-being and quality of life impact of the pandemic may be exacerbated in these groups due to pre-existing vulnerabilities. Lastly, we did not ask participants who chose “other” as their gender to specify their gender identity. However, considering that the data included only 21 participants, we do not believe it impacted the results or their interpretation substantially.

To conclude, while physical distancing measures and stay-at-home orders represent essential public health strategies for curbing the spread of the COVID-19 pandemic, they may be a severe threat to the psychological well-being of the general population. Using PROMs to assess psychological well-being and quality of life to monitor, plan and deliver healthcare resources should be an essential part of the COVID-19 response.

## Data Availability

The datasets used and/or analyzed during the current study are available from the corresponding author on reasonable request.

## References

[1] Cheung Y, Chau PH, Yip PS (2008). A revisit on older adults suicides and Severe Acute Respiratory Syndrome (SARS) epidemic in Hong Kong. Int J Geriatr Psychiatry J Psychiatry Late Life Allied Sci.

[2] Dsouza DD, Quadros S, Hyderabadwala ZJ, Mamun MA (2020). Aggregated COVID-19 suicide incidences in India: fear of COVID-19 infection is the prominent causative factor. Psychiatry Res.

[3] Gunnell D, Appleby L, Arensman E, Hawton K, John A, Kapur N (2020). Suicide risk and prevention during the COVID-19 pandemic. Lancet Psychiatry.

[4] Tucci V, Moukaddam N, Meadows J, Shah S, Galwankar SC, Kapur GB (2017). The forgotten plague: psychiatric manifestations of Ebola, Zika, and emerging infectious diseases. J Glob Infect Dis.

[5] Gao J, Zheng P, Jia Y, Chen H, Mao Y, Chen S (2020). Mental health problems and social media exposure during COVID-19 outbreak. PLoS ONE.

[6] Garfin DR, Silver RC, Holman EA (2020). The novel coronavirus (COVID-2019) outbreak: amplification of public health consequences by media exposure. Health Psychol.

[7] Black N (2013). Patient reported outcome measures may transform healthcare. BMJ (Overseas and Retired Doctors ed).

[8] Shi L, Lu Z-A, Que J-Y, Huang X-L, Liu L, Ran M-S (2020). Prevalence of and risk factors associated with mental health symptoms among the general population in China during the coronavirus disease 2019 pandemic. JAMA Netw Open.

[9] Wang C, Pan R, Wan X, Tan Y, Xu L, McIntyre RS (2020). A longitudinal study on the mental health of general population during the COVID-19 epidemic in China. Brain Behav Immunity.

[10] Qiu J, Shen B, Zhao M, Wang Z, Xie B, Xu Y (2020). A nationwide survey of psychological distress among Chinese people in the COVID-19 epidemic: implications and policy recommendations. Gen Psychiatry.

[11] Lopes BCdS, Jaspal R (2020). Understanding the mental health burden of COVID-19 in the United Kingdom. Psychol Trauma Theory Res Pract Policy.

[12] Burn W, Mudholkar S (2020). Impact of COVID-19 on mental health: Update from the United Kingdom. Indian J Psychiatry.

[13] Rossi R, Socci V, Talevi D, Mensi S, Niolu C, Pacitti F (2020). COVID-19 pandemic and lockdown measures impact on mental health among the general population in Italy. Front Psychiatry.

[14] Rodríguez-Rey R, Garrido-Hernansaiz H, Collado S (2020). Psychological impact and associated factors during the initial stage of the coronavirus (COVID-19) pandemic among the general population in Spain. Front Psychol.

[15] Goularte JF, Serafim SD, Colombo R, Hogg B, Caldieraro MA, Rosa AR (2020). COVID-19 and mental health in Brazil: psychiatric symptoms in the general population. J Psychiatr Res.

[16] Palan S, Schitter C (2018). Prolific.ac—a subject pool for online experiments. J Behav Exp Finance.

[17] Peer E, Brandimarte L, Samat S, Acquisti A (2017). Beyond the Turk: alternative platforms for crowdsourcing behavioral research. J Exp Soc Psychol.

[18] Prolific. https://www.prolific.co.

[19] De Souza JA, Yap BJ, Hlubocky FJ, Wroblewski K, Ratain MJ, Cella D (2014). The development of a financial toxicity patient-reported outcome in cancer: the COST measure. Cancer.

[20] de la Vega PB, Losi S, Martinez LS, Bovell-Ammon A, Garg A, James T (2019). Implementing an EHR-based screening and referral system to address social determinants of health in primary care. Med Care.

[21] Lam KH, Kwa VI (2018). Validity of the PROMIS-10 Global Health assessed by telephone and on paper in minor stroke and transient ischaemic attack in the Netherlands. BMJ Open.

[22] Suriani RJ, Kassam HF, Passarelli NR, Esparza R, Kovacevic D (2020). Validation of PROMIS Global-10 compared with legacy instruments in patients with shoulder instability. Should Elb.

[23] Nicholson AD, Kassam HF, Pan SD, Berman JE, Blaine TA, Kovacevic D (2019). Performance of PROMIS Global-10 compared with legacy instruments for rotator cuff disease. Am J Sports Med.

[24] Shim J, Hamilton D (2019). Comparative responsiveness of the PROMIS-10 Global Health and EQ-5D questionnaires in patients undergoing total knee arthroplasty. Bone Joint J.

[25] Hays RD, Bjorner JB, Revicki DA, Spritzer KL, Cella D (2009). Development of physical and mental health summary scores from the patient-reported outcomes measurement information system (PROMIS) global items. Qual Life Res.

[26] Cella D, Riley W, Stone A, Rothrock N, Reeve B, Yount S (2010). The Patient-Reported Outcomes Measurement Information System (PROMIS) developed and tested its first wave of adult self-reported health outcome item banks: 2005–2008. J Clin Epidemiol.

[27] Hays RD, Spritzer KL, Thompson WW, Cella D (2015). US general population estimate for “excellent” to “poor” self-rated health item. J Gen Intern Med.

[28] Spitzer RL, Kroenke K, Williams JB, Löwe B (2006). A brief measure for assessing generalized anxiety disorder: the GAD-7. Arch Intern Med.

[29] Löwe B, Decker O, Müller S, Brähler E, Schellberg D, Herzog W (2008). Validation and standardization of the Generalized Anxiety Disorder Screener (GAD-7) in the general population. Med Care.

[30] Kroenke K, Spitzer RL, Williams JB, Monahan PO, Löwe B (2007). Anxiety disorders in primary care: prevalence, impairment, comorbidity, and detection. Ann Intern Med.

[31] Johnson SU, Ulvenes PG, Øktedalen T, Hoffart A (2019). Psychometric properties of the GAD-7 in a heterogeneous psychiatric sample. Front Psychol.

[32] Kertz S, Bigda-Peyton J, Bjorgvinsson T (2013). Validity of the Generalized Anxiety Disorder-7 Scale in an acute psychiatric sample. Clin Psychol Psychother.

[33] Gilbody S, Richards D, Brealey S, Hewitt C (2007). Screening for depression in medical settings with the Patient Health Questionnaire (PHQ): a diagnostic meta-analysis. J Gen Intern Med.

[34] Kroenke K, Spitzer RL (2002). The PHQ-9: a new depression diagnostic and severity measure. Psychiatr Ann.

[35] Team RC. R: a language and environment for statistical computing. 2013.

[36] Zhao Q, Sun X, Xie F, Chen B, Wang L, Hu L (2021). Impact of COVID-19 on psychological wellbeing. Int J Clin Health Psychol.

[37] Solomou I, Constantinidou F (2020). Prevalence and predictors of anxiety and depression symptoms during the COVID-19 pandemic and compliance with precautionary measures: age and sex matter. Int J Environ Res Public Health.

[38] Smith L, Jacob L, Yakkundi A, McDermott D, Armstrong NC, Barnett Y (2020). Correlates of symptoms of anxiety and depression and mental wellbeing associated with COVID-19: a cross-sectional study of UK-based respondents. Psychiatry Res.

[39] Rossell SL, Neill E, Phillipou A, Tan EJ, Toh WL, Van Rheenen TE (2021). An overview of current mental health in the general population of Australia during the COVID-19 pandemic: results from the COLLATE project. Psychiatry Res.

[40] French MT, Mortensen K, Timming AR (2020). Psychological distress and coronavirus fears during the initial phase of the covid-19 pandemic in the United States. J Ment Health Policy Econ.

[41] Gloster AT, Lamnisos D, Lubenko J, Presti G, Squatrito V, Constantinou M (2020). Impact of COVID-19 pandemic on mental health: an international study. PLoS ONE.

[42] Zhou Y, MacGeorge EL, Myrick JG (2020). Mental health and its predictors during the early months of the COVID-19 pandemic experience in the United States. Int J Environ Res Public Health.

[43] Haliwa I, Wilson J, Lee J, Shook NJ (2021). Predictors of change in mental health during the COVID-19 pandemic. J Affect Disord.

[44] Chen K-Y, Li T, Gong F-H, Zhang J-S, Li X-K (2020). Predictors of health-related quality of life and influencing factors for COVID-19 patients, a follow-up at one month. Front Psychiatry.

[45] Duan H, Yan L, Ding X, Gan Y, Kohn N, Wu J (2020). Impact of the COVID-19 pandemic on mental health in the general Chinese population: changes, predictors and psychosocial correlates. Psychiatry Res.

[46] Özdin S, Bayrak ÖŞ (2020). Levels and predictors of anxiety, depression and health anxiety during COVID-19 pandemic in Turkish society: the importance of gender. Int J Soc Psychiatry.

[47] Naser AY, Dahmash EZ, Al-Rousan R, Alwafi H, Alrawashdeh HM, Ghoul I (2020). Mental health status of the general population, healthcare professionals, and university students during 2019 coronavirus disease outbreak in Jordan: a cross-sectional study. Brain Behav.

[48] Malesza M, Kaczmarek MC (2021). Predictors of anxiety during the COVID-19 pandemic in Poland. Pers Individ Differ.

[49] Verdolini N, Amoretti S, Montejo L, García-Rizo C, Hogg B, Mezquida G (2021). Resilience and mental health during the COVID-19 pandemic. J Affect Disord.

[50] Xiong J, Lipsitz O, Nasri F, Lui LM, Gill H, Phan L (2020). Impact of COVID-19 pandemic on mental health in the general population: a systematic review. J Affect Disord.

[51] Vindegaard N, Benros ME (2020). COVID-19 pandemic and mental health consequences: systematic review of the current evidence. Brain Behav Immun.

[52] Ogbodo JN, Onwe EC, Chukwu J, Nwasum CJ, Nwakpu ES, Nwankwo SU (2020). Communicating health crisis: a content analysis of global media framing of COVID-19. Health Promot Perspect.

[53] Drouin M, McDaniel BT, Pater J, Toscos T (2020). How parents and their children used social media and technology at the beginning of the COVID-19 pandemic and associations with anxiety. Cyberpsychol Behav Soc Netw.

[54] Lei L, Huang X, Zhang S, Yang J, Yang L, Xu M (2020). Comparison of prevalence and associated factors of anxiety and depression among people affected by versus people unaffected by quarantine during the COVID-19 epidemic in Southwestern China. Med Sci Monit Int Med J Exp Clin Res.

[55] Mazza C, Ricci E, Biondi S, Colasanti M, Ferracuti S, Napoli C (2020). A nationwide survey of psychological distress among Italian people during the COVID-19 pandemic: immediate psychological responses and associated factors. Int J Environ Res Public Health.

[56] Ng KH, Agius M, Zaman R (2013). The global economic crisis: effects on mental health and what can be done. J R Soc Med.

[57] Norton A, Olliaro P, Sigfrid L, Carson G, Hastie C, Kaushic C (2021). Long COVID: tackling a multifaceted condition requires a multidisciplinary approach. Lancet Infect Dis.

[58] Iqbal FM, Lam K, Sounderajah V, Elkin S, Ashrafian H, Darzi A (2021). Understanding the survivorship burden of long COVID. EClinicalMedicine.

[59] Wong AW, Shah AS, Johnston JC, Carlsten C, Ryerson CJ (2020). Patient-reported outcome measures after COVID-19: a prospective cohort study. Eur Respir J.

[60] Aiyegbusi OL, Calvert MJ (2020). Patient-reported outcomes: central to the management of COVID-19. Lancet (London, England).

